# Design of a Trustworthy Cloud-Native National Digital Health Information Infrastructure for Secure Data Management and Use

**DOI:** 10.1093/oodh/oqae043

**Published:** 2024-11-03

**Authors:** John Kar-Kin Zao, Joseph Tsung-Shu Wu, Kennedy Kanyimbo, Florian Delizy, Tchin-Tze Gan, Hung-I Kuo, Chih-Hao Hsia, Chuan-Hsi Lo, Shang-Hui Yang, Clément Jean Alexandre Richard, Billy Rajab, Maganizo Monawe, Blessings Kamanga, Nikotelhe Mtambalika, Kwong-Leung Joseph Yu, Chih-Feng Chou, Choo-Aun Neoh, Joseph Gallagher, John O'Donoghue, Rebecca Mtegha, Hsin-Yi Lee, Alinafe Mbewe

**Affiliations:** FiduciaEdge Technologies Co. Ltd., Hsinchu City, Taiwan, R.O.C; Digital Health Division (DHD), Malawi Ministry of Health, Lilongwe, Malawi; Luke International, Nøtterøy, Norway; Pingtung Christian Hospital, Pingtung City, Taiwan, R.O.C; Digital Health Division (DHD), Malawi Ministry of Health, Lilongwe, Malawi; FiduciaEdge Technologies Co. Ltd., Hsinchu City, Taiwan, R.O.C; College of Electrical Engineering and Computer Science (EECS), National Yang Ming Chiao Tung University, Hsinchu City, Taiwan, R.O.C; Graduate School of Engineering, Katahira Campus, Tohoku University, Sendai, Japan; FiduciaEdge Technologies Co. Ltd., Hsinchu City, Taiwan, R.O.C; FiduciaEdge Technologies Co. Ltd., Hsinchu City, Taiwan, R.O.C; WiAdvance Technology Co. Ltd., New Taipei City, Taiwan, R.O.C; WiAdvance Technology Co. Ltd., New Taipei City, Taiwan, R.O.C; WiAdvance Technology Co. Ltd., New Taipei City, Taiwan, R.O.C; FiduciaEdge Technologies Co. Ltd., Hsinchu City, Taiwan, R.O.C; Digital Health Division (DHD), Malawi Ministry of Health, Lilongwe, Malawi; Digital Health Division (DHD), Malawi Ministry of Health, Lilongwe, Malawi; Digital Health Division (DHD), Malawi Ministry of Health, Lilongwe, Malawi; Luke International, Nøtterøy, Norway; Digital Health Division (DHD), Malawi Ministry of Health, Lilongwe, Malawi; Luke International, Nøtterøy, Norway; Luke International, Nøtterøy, Norway; Pingtung Christian Hospital, Pingtung City, Taiwan, R.O.C; Pingtung Christian Hospital, Pingtung City, Taiwan, R.O.C; Pingtung Christian Hospital, Pingtung City, Taiwan, R.O.C; University College Dublin, Dublin, Ireland; University College Cork, Cork, Ireland; Malawi eHealth Research Center, Mzuzu University, Mzuzu, Malawi; Luke International, Nøtterøy, Norway; Luke International, Nøtterøy, Norway; Digital Health Division (DHD), Malawi Ministry of Health, Lilongwe, Malawi

**Keywords:** National Digital Health Information System, health information security, Internet of Medical Things, cloud-native computing, Zero-Trust Architecture, Trusted Computing, Iso27001

## Abstract

Since 2022, Malawi Ministry of Health (MoH) designated the development of a National Digital Health Information System (NDHIS) as one of the most important pillars of its national health strategy. This system is built upon a distributed computing infrastructure employing the following state-of-art technologies: (i) digital healthcare devices to capture medical data; (ii) Kubernetes-based Cloud-Native Computing architecture to simplify system management and service deployment; (iii) Zero-Trust Secure Communication to protect confidentiality, integrity and access rights of medical data transported over the Internet; (iv) Trusted Computing to allow medical data to be processed by certified software without compromising data privacy and sovereignty. Trustworthiness, including reliability, security, privacy and business integrity, of this system was ensured by a peer-to-peer network of trusted medical information guards deployed as the gatekeepers of the computing facility on this system. This NDHIS can facilitate Malawi to attain universal health coverage by 2030 through its scalability and operation efficiency. It shall improve medical data quality and security by adopting a paperless approach. It will also enable MoH to offer data rental services to healthcare researchers and AI model developers around the world. This project is spearheaded by the Digital Health Division (DHD) under MoH. The trustworthy computing infrastructure was designed by a taskforce assembled by the DHD in collaboration with Luke International in Norway, and a consortium of hardware and software solution providers in Taiwan. A prototype that can connect community clinics with a district hospital has been tested at Taiwan Pingtung Christian Hospital.

## INTRODUCTION

Located in Sub-Saharan Africa, Malawi is one of the developing countries that is aggressively implementing the UN Sustainable Development Goals (SDGs) in good health, quality education, gender equality, clean water, sustainable cities, climate actions, and strong institutions [1]. To enable their citizens in attaining good health and well-being (SDG #3), Malawi strives to provide universal health coverage (UHC, Target 3.8) in both urban and suburban areas in the country [2]. Following a recommendation from the World Health Organization (WHO) [3], the Malawi Ministry of Health (MoH) designated the development of a sustainable and harmonized country-led National Digital Health Information System (NDHIS) as one of the most important pillars of its national health strategy and an essential means to track its UHC progress. According to Malawi Health Sector Strategic Plan III (HSSP-III) [4], the NDHIS shall empower Malawi to attain UHC by 2030 [1].

Unfortunately, poor data quality, significant security vulnerability and notable service fragmentation are hindering the realization of this system (Overview of Malawi National Digital Health Plan section). A taskforce was thus established in early 2023 to investigate state-of-art information technologies and security practices including handheld digital healthcare devices, distributed Cloud-Native Computing and Trusted Computing to develop a trustworthy^1^ National Digital Health Information Infrastructure (T-NDHII) as communication and computing infrastructure for running the NDHIS. This taskforce was assembled by the Digital Health Division (DHD) under the Malawi MoH in collaboration with Luke International in Norway and a consortium of hardware and software solution providers including FiduciaEdge Technologies and WiAdvance Technology in Taiwan. This paper presents the T-NDHII architecture proposed by this taskforce. The proposed architecture hinges on the use of one type of security element, known as the *trusted Medical Information Guards*, to protect the transportation and processing of digital health data at all levels of the NDHIS. This infrastructure, combined with proper security certification of the computing equipment and their operating sites as well as strict compliance to the standard information security practices, shall overcome the security challenges and facilitate Malawi to achieve its UHC goal.

### Outline of this paper

This paper comprises seven sections. This section briefly outlines the history of digital health services in Malawi, setting the stage for the current effort. In the Objective section, we explain the vision and strategy behind the NDHIS and review the challenges that prompted the design of T-NDHII. The Method section describes the four design principles and the tMIG-centric architecture of T-NDHII, linking each principle to the corresponding challenges. We also examine the reliability and scalability of this security architecture. The Result section details the implementation of the OpenHIE-based NDHIS at community, facility, district, and national levels, introducing the Integrated Community Health Information System (iCHIS) and the Malawi Health Information System (MaHIS). The Use Case section presents two cases demonstrating the T-NDHII architecture’s capabilities: a pilot project at Taiwan Pingtung Christian Hospital showcasing secure inter-facility information exchange, and the BIOTOPE project in Ireland for identifying children at risk of severe pneumonia in Malawi demonstrating the potential offering of *trusted data leasing* services [5] to research and commercial partners through T-NDHII. Finally, the Discussion section outlines a timeline for deploying the T-NDHII. This paper was concluded with a summary of its unique features and its role in the Malawi Digital Health Strategy.

### History of digital healthcare in Malawi

**Figure 1:**
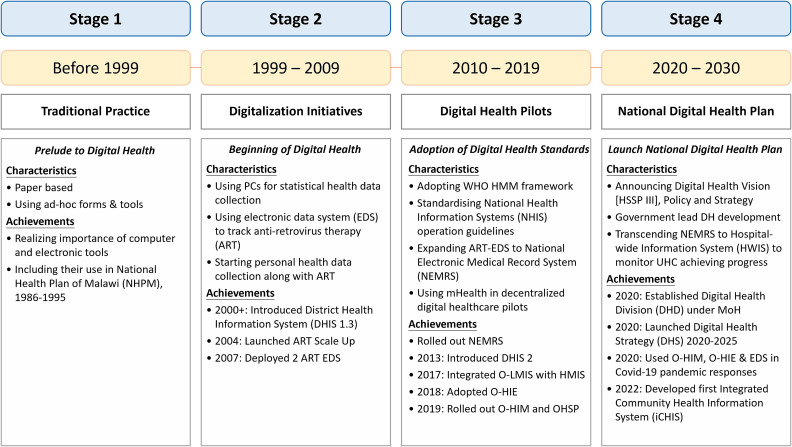
Development stages of Malawi Health Information Infrastructure

As shown in Fig. 1), Malawi’s journey to digital healthcare began at the dawn of the 21st century and can be divided into four stages. Before 1999, hospitals in the entire country keep paper health records in ad-hoc forms. However, the MoH already realized the importance of using computers to manage patients’ health records and included computerization as a goal in the National Health Plan of Malawi, 1986–1995 [6].

Malawi began to adopt digital healthcare technologies in the 2000s. It started with the collection of statistical health data using the District Health Information Software (DHIS 1.3) system installed in the central hospitals and the district health offices. This system eventually became the nation’s Health Management Information System (HMIS) through a series of upgrades including: the adoption of DHIS 2 in 2013, the development of the One Health Surveillance Platform (OHSP) in 2020 [7] and the integration with Open Logistics Management Information System (O-LMIS) in 2021 [8]. In response to the AIDS pandemic, Malawi joined the national antiretroviral therapy (ART) scale-up in 2004 and deployed two ART electronic data systems with US-CDC funding in 2007. These ART-EDS later evolved into a National Electronic Medical Records System (NEMRS) for collecting individual health data. This system eventually became the bedrock of the MaHIS, which will be installed in all district and regional healthcare facilities by 2027 (Malawi Health Information System section).

In the 2010s, Malawi began to follow the standard eHealth and mHealth practices. It adopted the World Health Organization Health Metrics Network (WHO-HMN) framework as the blueprint of the nation’s health information infrastructure. Then, in 2018, it adopted the Open Health Information Exchange (OpenHIE) as the common platform of the NDHIS. This strategic decision enabled the NDHIS and the underlying T-NDHII to employ the state-of-art Cloud-Native Computing and Trusted Computing technologies due to the use of software containers to encapsulate all OpenHIE modules.

Starting from 2020, Malawi MoH has taken bold steps to lay out a national digital healthcare plan and develop an NDHIS. It established the DHD under MoH in 2020 and announced the Digital Health Strategy [9] in the same year. In 2021, DHD utilizes all its digital health arsenals to tackle the COVID-19 pandemic and became one of the pioneering countries that took the digital health approaches to monitor and control the pandemic [10]. In 2022, DHD deployed the first iCHIS. From 2024 to 2030, DHD plans to develop the NDHIS and the underlying T-NDHII with funding from various development partners.

In the past two decades, the Central Monitoring and Evaluation Division (CMED) and the DHD under Malawi MoH have been the key organizations responsible for leading the NDHIS development projects. Long-standing development partners also include the US-CDC, the USAID, the Bill and Melinda Gates Foundation, the Global Fund, the World Bank, UNICEF, WHO, UNDP, GIZ, NORAD, DFID and GAVI.

### State of digital healthcare in Malawi

**Figure 2:**
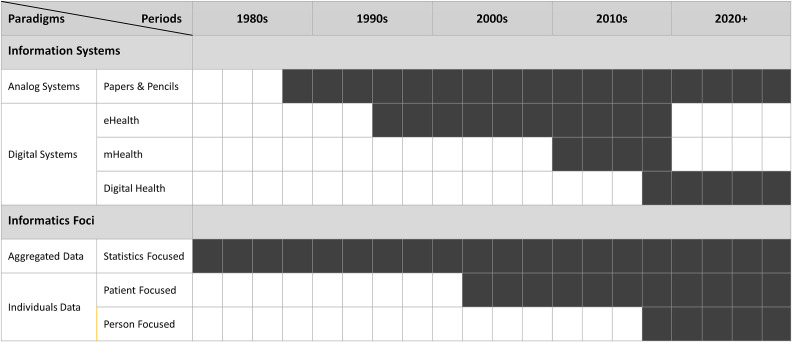
Paradigm shifts in Malawi National Health Information Systems

Like all evolving services, the healthcare services in Malawi were delivered using a variety of legacy systems (Fig. 2). Even nowadays, a significant portion of district hospitals and community clinics, especially those in the rural areas, are still using paper health records. This can result in poor data quality, improper data disclosure and inadvertent data loss. It will take time and effort to replace them with a National Electronic Medical Record System (NEMRS). Moreover, legacy eHealth and mHealth systems need to be integrated with the newly adopted OpenHIE system. Nevertheless, two decades of investment and development effort have notably improved and extended the healthcare services in Malawi. When the NDHIS is deployed, it will help the country to achieve its UHC goal.

## OBJECTIVE

In this section, we survey the objectives and the implementation strategies of the Malawi National Digital Health Plan as well as the challenges encountered while implementing this plan. These objectives, strategies and challenges provided a basis for the T-NDHII taskforce to formulate the design principles and propose the system architecture mentioned in the Method section.

### Overview of Malawi National Digital Health Plan

Between 2020 and 2023, Malawi MoH published three documents to specify its vision, strategy and policy to implement the national digital health plan:

1. *Vision, HSSP III (2023–2030)* [4]: to empower all Malawians to achieve a state of health that will enable them to lead a quality and productive life.
2. *Strategy, Digital Health Strategy (2020–2025)* [9]: to develop a sustainable and harmonized country-led digital health system to enable efficient delivery of health services to beneficiaries at all levels.
3. *Policy, Digital Health Policy (2023–2027)* [11]: to provide direction on the key issues central to development, utilization and functioning of digital health solutions and service delivery.

As listed in Table 1, the Malawi Digital Health Strategy (DHS) established the following seven objectives and their empowering strategies^2^ to fulfil the UN-SDG #3 on good health. Among them, objectives 4–7 are directly related to the T-NDHII taskforce’s mandate to improve *data quality, accessibility* and *security* for NDHIS.

**Table 1:** Malawi digital health strategy objectives and empowering strategies

**Objective 1: Improve coordination of digital health investments.**
Strategy 1.1 Strengthen leadership and governance structures.
Strategy 1.2 Create environments for effective implementation of digital health solutions.
Strategy 1.3 Strengthen sustainability mechanisms for digital health solutions.
**Objective 2: Establish reliable ICT infrastructure for utilizing digital health systems.**
Strategy 2.1 Improve connectivity in poorly covered areas.
Strategy 2.2 Extend coverage of renewable and hybrid power solutions.
**Objective 3: Build communities and health worker capacity to participate in digital health.**
Strategy 3.1 Increase health workers’ capacity to utilize digital health solutions.
**Objective 4: Leverage technology to increase access to and quality of health service delivery.**
Strategy 4.1 Use innovative digital health technologies to increase digital health service access.
Strategy 4.2 Use big data and predictive analytics to improve health services.
Strategy 4.3 Align digital health interventions with comprehensive digital health architecture.
**Objective 5: Improve security of digital health information and systems.**
Strategy 5.1 Ensure continuity of service delivery at all points of service.
Strategy 5.2 Implement standardized security management process.
Strategy 5.3 Enforce privacy and security protection on patient data.
Strategy 5.4 Protect digital health systems from security threats including physical intrusion, malwares attacks, frauds, theft and privacy breaching.
**Objective 6: Promote continuity of care through shared health records.**
Strategy 6.1 Ensure longitudinal and cross-sectional accessibility of patients’ health records.
Strategy 6.2 Strengthen identification of patients and staff.
**Objective 7: Strengthen data accessibility and sharing across systems.**
Strategy 7.1 Implement a standard framework on national level to ensure interoperability. — Enable digital health systems to share data based on the OpenHIE Framework.
Strategy 7.2 Implement interoperability among priority systems including DHIS2 and Open LMIS, LIMS, EMRs, CRVS and mHealth applications.

### Challenges in existing system and practice

The primary obstacles to achieving the DHS objectives have emerged as existing challenges, particularly in information and service management, as discussed in this section. Addressing these challenges drives the development of the T-NDHII design principles outlined in the Design Principles section.

#### Poor data quality

##### Cause 1. Widespread use of paper health records

In Malawi countryside, where the National Electronic Medical Record System (NEMRS) is unavailable, patients’ personal health records (known as their health passports) are kept in paper files carried by the patients. These records are then entered into the Health Management Information System (HMIS) through DHIS2 only after they are brought to the HMIS officer in a district health clinic or a regional hospital after long delays. Throughout this process, the patients’ personal health records may be entered erroneously, damaged, or missing. Using digital healthcare devices instead of paper records to collect and maintain patients’ health information can mitigate this issue. This approach aligns with DHS Objective 4 and is addressed by Design Principle 1 (Principle 1: use of digital healthcare devices section).

##### Cause 2. Poor training of healthcare workers

The healthcare workers may not be well trained to accurately record patients’ conditions. This issue aligns with DHS Objective 3 and can be mitigated by providing healthcare workers with pre-service digital healthcare training, followed by continuous professional development through online learning [12] and in-person workshops.

##### Cause 3. Poor Internet connectivity in rural areas

Internet connections and even electric power are often unavailable for setting up a NEMRS point of service in the rural areas. This issue aligns with DHS Objective 2 and will be addressed through the development of a new generation of the iCHIS POS (Integrated Community Health Information System section).

#### Significant data leakage

##### Cause 1. Paper record leakage

Since patients’ health data records are mostly kept in papers, unauthorized personnel can easily access those records without proper clearance. The leaked personal health data, such as the patients’ HIV-related history, are confidential and private in nature. As stated in Design Principle 1 (Principle 1: use of digital healthcare devices section), data leakage can be prevented with the use of digital healthcare devices instead of paper health records.

##### Cause 2. Poor access control

Although the health record access protocols have been established at the national level, these protocols have not yet been enforced among the district-level facilities. For example, although NEMRS have username and password-based access control, healthcare workers sometimes share their credentials to access the system. The mitigation of this cause correlates with DHS Objective 6. According to Design Principle 4B, this issue will be addressed by issuing Health Worker Identities (HWIDs) to healthcare workers, enabling them to properly authenticate themselves to NEMIS (Healthcare worker identification and access control to digital health information systems section).

#### Insufficient security protection

##### Cause 1. Site vulnerability

The server rooms on the government premises have not satisfied the information security management standards. Computing equipment installed in these server rooms can thus be exposed to various physical and operational security attacks. This issue aligns with DHS Objective 5 and will be addressed by enforcing ISO27001 compliance certification on all T-DNHII equipment sites, as outlined in Design Principle 4A (Security certification of NDHII equipment sites section).

##### Cause 2. Platform vulnerability

The computing servers installed in district-, regional- and national-level facilities are not adequately protected from cybersecurity attacks. Ransomware and denial-of-service attacks have occurred repeatedly ever since the deployment of DHIS2 and NEMIS systems. This issue also aligns with DHS Objective 5 and will be addressed by enforcing Trusted Computing practices on all computing equipment hosting NDHIS services, as outlined in Design Principle 3 (Principle 3: implementation of trusted computing practice section).

##### Cause 3. Software vulnerability

The open-source healthcare software applications have not gone through vulnerability analyses or safety checks. The mitigation of this cause also correlates with DHS Objective 5. This issue will be addressed by applying Design Principle 3C (Secure system boot and software certification section), which mandates that all software running on T-NDHII must provide their software bills of materials (SBOM) and passes static application security tests (SAST) with no critical CVE vulnerabilities [13].

#### Fragmentation of digital healthcare services

##### Problem scenarios

The digital healthcare landscape in Malawi exhibits disparity in the maturity levels of different services due to the concentration of investments in specific domains. For instance, considerable investments have been allocated to establish information systems tailored for HIV-related services while limited attention has been given to the needs of other healthcare services.

This issue aligns with DHS Objective 1. From a governance perspective, it requires better coordination within MoH DHD and the creation of task forces, such as the T-NDHII taskforce, to address specific issues. From an engineering perspective, successfully deploying T-NDHII based on OpenHIE, with necessary customizations for Malawi, will be crucial to achieving this DHS objective.

## METHOD

With the aim of achieving DHS Objectives 4–7 by overcoming the challenges mentioned in the previous section, the T-NDHII taskforce recommended four design principles and proposed a system architecture that hinges on the use of a single cybersecurity element, the trusted Medical Information Guard (tMIG), and the adoption of Trusted Computing Practice.

### Design principles

#### Principle 1: use of digital healthcare devices

DHS Strategy 4.1 mandated the use of innovative digital health technologies to enhance accessibility and quality of digital health services in Malawi. The first step taken is to use digital healthcare devices in lieu of paper records to track patients’ conditions. The following list of electronic medical instruments will be attached to the next-generation iCHIS to help achieve this objective.

- digital thermometers and stethoscopes,
- blood pressure, peripheral capillary oxygen saturation (SpO2) and blood glucose monitors,
- body weight and composition analyzers (a.k.a. bioelectrical impedance analyzers),
- handheld digital endoscope such as the MiiS Horus Scope [14],
- handheld ultrasound scanners such as the ASUS Handheld Ultrasound Solution LU800 [15],
- portable digital X-ray machine such as the ZiMed Digital Portable X RAY ZPXR-A11 [16].

The chosen devices are handheld or portable, easy to use and robust in their operation. As described in the Integrated Community Health Information System section, they will be connected to a trusted mini-Medical Information Guard (mini-tMIG) that will support Bluetooth Generic Health Sensor (GHS) Profile [17] for real-time interactive remote patient monitoring. Through Zero-Trust Secure Communication, the mini-tMIG will conduct authorized information exchanges with the Malawi Healthcare Information System (MaHIS) installed in the district/regional hospitals and ultimately with the digital health services hosted in the National Health Data Centers (NHDC).

#### Principle 2: adoption of distributed cloud-native computing architecture

##### Deployment of containerized healthcare applications

In 2018, Malawi MoH adopted Open Health Information Exchange (OpenHIE) as the common platform for its NDHIS. This decision dictated that the health information services offered at all levels of NDHIS will hence be implemented as software containers, which are standardized package of program codes and libraries that can run reliably on different computers on demand without modification [18]. This state-of-the-art technology enables health information services to be deployed quickly onto computers installed in district-, regional- and national-level facilities using automated procedures.

##### Distributed information processing among field equipment and data centers

To fully align with the OpenHIE framework, the MoH utilizes Kubernetes to manage containerized health information services running on the NDHIS. The underlying T-NDHII must therefore operate as a distributed Edge-Cloud Computing infrastructure with the community-based iCHIS and hospital-based MaHIS operating at the Edge while the National Health Data Centers (NDHC) function as the Cloud. The T-NDHII also operates as a Cloud-Native Computing framework [19], which enables the entire computing infrastructure to operate without disruption throughout incremental deployment and hardware/software updates. Certified hardware equipment can be added to scale up its capability and coverage. Verified containerized software can also be deployed on demand through Kubernetes and over-the-air updates to implement new functions.

#### Principle 3: implementation of trusted computing practice

Since Trusted Computing demands high assurance in *reliability* of computing platforms, *security* of information exchange and storage and *privacy preservation* in data processing, the T-NDHII architecture must therefore enforce the following three essential practices.

##### Zero-Trust Network Security

Proposed by the USA National Institute of Standards and Technology (NIST) [20], the Zero-Trust Architecture (ZTA) is the recommended *information security practice* in the era of mobile communication and Internet of Things. It consists of the following three practices:

1. *Confidentiality and integrity protection of communications*—this practice corresponds to the recommended communication security protection of data confidentiality and integrity.
2. *Mutual authentication of communicating entities*—this practice requires cryptographically strong mutual authentication of both communicating devices and communicating parties (users or processes).
3. *Least privileged access of information*—this practice requires the enforcement of information access control based on the roles or attributes of the users or processes.

Trusted Medical Information Guards (tMIGs) (Trusted Medical Information Guards section) must be installed in front of every iCHIS, MaHIS and NHDC as their gatekeepers to enforce ZTA policies on all information interchanges occurred in the T-NDHII architecture. The tMIGs will be delivered as turn-key systems ready for field deployment. An international healthcare system integrator will be chosen by MoH to deploy these systems and configure the tMIG network.

##### Multiple Independent Levels of Computing Security

Besides the enforcement of Zero-Trust Network Security, T-NDHII also adopts the principles of Multiple Independent Levels of Security (MILS) [21] to protect computation security and data privacy. The essence of MILS is to separate computing processes and information flows through (i) partition of computing environment, (ii) controlled usage of computing resources and (iii) controlled communication between computing processes.

In T-NDHII, the three MILS functions are implemented by the Trusted Rich Execution Environments (T-REEs) [22] instantiated in every tMIG. Each T-REE can:

1. Use kernel-level isolation among container pods to separate their computing environments.
2. Use mandatory access control of memory, I/O and storage to control resource usage by pods.
3. Use capability-based authorization to control communication between the authorized pods.

Each container pod will run in its own T-REE. The computing security environment of each T-REE will be configured through Kubernetes container lifecycle management before the container runs in the pod. Hence, no modification to the program codes will ever be needed.

##### Secure system boot and software certification

As a basic requirement of Trusted Computing, every piece of computing equipment in the T-NDHII must possess its own unique (public key-based) security credentials and support secure booting of their system firmware and software.

Furthermore, to ensure that the application software running in the T-NDHII will not succumb to cyberattacks, all applications running on the Edge and Cloud servers must provide their software bills of materials (SBOM) and pass static application security tests (SAST) with no critical CVE vulnerability [13]. All these applications must be run in T-REEs, which enforce information isolation and mandatory resource access control. In case of a cyberattack, the damage caused by the affected software will be contained within its own T-REE. The T-REE containing the affected software will be purged. A new T-REE with the original software will then be created to replace the purged one.

#### Principle 4: compliance to cybersecurity standards

##### Security certification of NDHII equipment sites

Operation of the National Health Data Center (NHDC) must be fully compliant with ISO27001 standard for information security management systems (ISMS) [23], including proper worker authentication and authorization for physical and online access of NHDC hardware and software.

##### Healthcare worker identification and access control to digital health information systems

The MoH is developing an integrated human resource information system (iHRIS) to serve as the single source for all health worker data. MoH will collaborate with the National Registration Bureau to issue Healthcare Worker identities (HWIDs) in the form of smart cards and electronic wallet tokens. These HWIDs will be used to authorize healthcare workers' access to the OpenHIE-based NDHIS.

### System architecture

The entire Malawi NDHIS is built upon the OpenHIE architecture. It is the mission of the T-NDHII taskforce to ensure trustworthiness of the entire infrastructure without altering the OpenHIE-based structure and the information flows of NDHIS.

In this section, we first review the layered architecture of OpenHIE and then describe an approach to ensure the trustworthiness of this architecture through the introduction of a single cybersecurity element, the *Trusted Medical Information Guard (tMIG)*, at its interoperability layer*.*

#### OpenHIE functional layers

**Figure 3:**
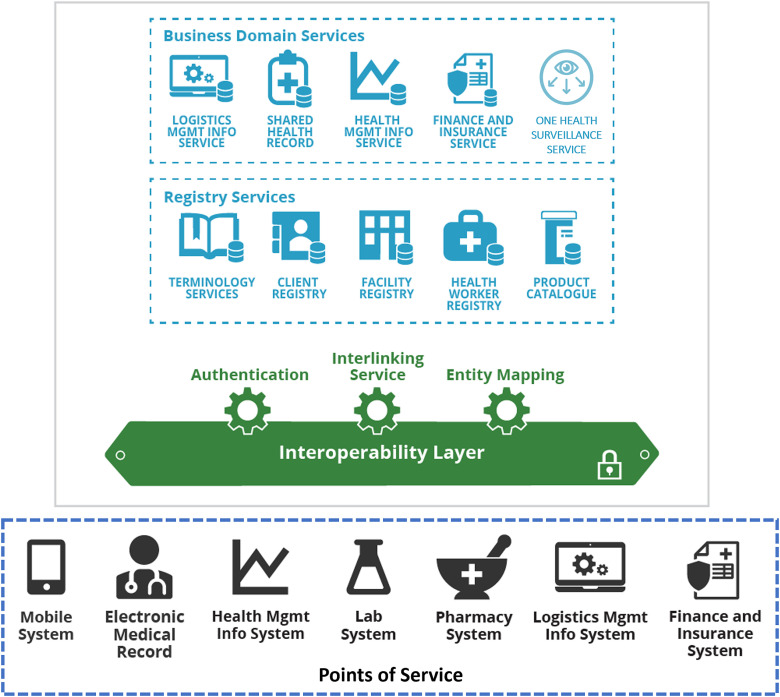
OpenHIE layered architecture

OpenHIE is a cloud-native health information system. All its components are implemented as containerized software modules managed using Kubernetes. The entire system can be divided into three functional layers (Fig. 3).

##### Point-of-Service (POS) Layer

This bottom layer consists of the sources and the sinks of health information including medical instruments, and the computerized physician order entry (CPOE) consoles, the health management information system (HMIS), the logistic management information systems (LMIS) and the finance/insurance systems. These Points of Services (POS) are the clients to the health information services provided on the top layer of the OpenHIE system.

##### Health Information Service (HIS) Layer

This top layer contains all the services necessary to run the NDHIS. It can be subdivided into a *Registry Service Layer* and a *Business Service Layer*. Using Kubernetes, OpenHIE can deploy these services in any data center, hospital servers and even field computers on demand. These services can communicate with one another and the POS through the interoperability layer.

##### Interoperability Layer (IL)

This middle layer is the most important component in OpenHIE. It monitors, routes, filters and transforms information passing between the POS and the HIS layers.

The OpenHIE IL is implemented with the Open Health Information Mediator (OpenHIM), a distributed system composed of containerized core modules running in OpenHIE computing nodes. OpenHIM passes information between its *clients* (POS and HIS) by establishing a *route* between them through a resource discovery service. Every OpenHIE computing node and its clients have unique identifiers. All transactions are conducted via RESTful web services and tracked by audit services to ensure HIPAA compliancy.

Besides performing message routing, OpenHIM can also run user-defined micro-services as Mediators to filter and transform its forwarded messages. High-availability services can be provided by connecting each OpenHIM core with multiple peers. Alternative routes and mediators can be used when the default ones become unavailable. Both OpenHIM Core and Mediator modules can be configured, monitored and managed by the OpenHIM Administrator running in the Cloud Servers.

#### Trusted Medical Information Guards

Information Guards are originally used in military to implement ‘electronic air gaps’ within a multiple level security (MLS) system [24]. More than firewalls, Guards work to limit the information exchanged between the communicating parties by performing data sanitization and application proxying in isolated computing environments. On the Internet, Guards are often installed as the bastion hosts [25] deployed between an institutional intranet and the public extranet.

The trusted Medical Information Guard (tMIG) used to protect the Malawi T-NDHII is specially designed to encapsulate an OpenHIM Core and its associated Mediators in a Trusted Computing environment enforcing Zero-Trust Networking (Zero-Trust Network security section) and Multiple Independent Levels of Security (Multiple Independent Levels of Computing Security section). By deploying these tMIGs as the security frontends at the community-, district-, regional- and national-level facilities and observing the secure operation procedures specified in the Secure system boot and software certification, the Security certification of NDHII equipment sites and the Healthcare worker identification and access control to digital health information systems sections, the T-NDHII will be free from the security vulnerability and data leakage problems described in the Challenges in existing system and practice section.

**Figure 4:**
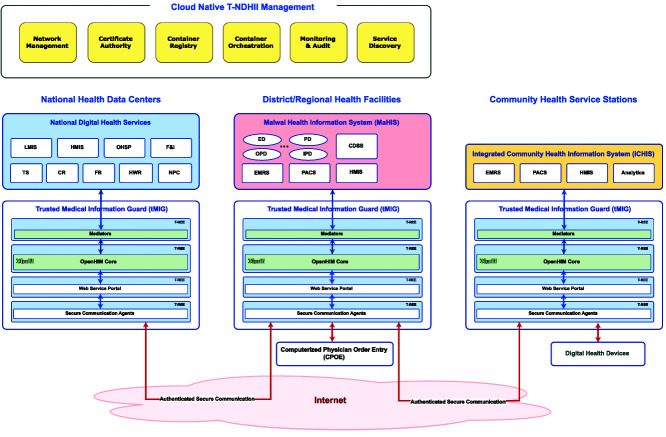
System architecture of Malawi Trustworthy T-NDHII with tMIG protection at the community, district/regional, and national levels

As shown in Fig. 4, each tMIG includes four essential components:

***OpenHIM core:*** performing message routing and transaction monitoring on each and every data channel.

***OpenHIM mediators:*** attached to each data channel to perform data filtering and transformation. T-NDHII currently provides secure channels for HL7/FHIR, DICOMweb and DHIS2/ADX data transfers.

***Web service portal:*** built into the tMIG to provide RESTful web service and web browser interfaces to different POS. With proper authorization, cell phones and tablets can access health information services through a webpage provided by tMIG.

***Secure communication agents:*** Each tMIG enforces Zero-Trust Networking by using a valid public key-based LDevID credential, which it obtained during installation, to perform mutual authentication with all its communicating peers and the POS. Data confidentiality and integrity protection may also be provided through virtual private networks (VPN) and Secure HTTP connections.

The services offered in the tMIGs are arranged into a dataflow pipeline starting with secure communication and web-based access to message routing and transformation. The entire tMIG software system will run on a trusted Edge Computing platform that can provide information isolation among container pods running in the Trusted Rich Execution Environments (T-REE) [22].

#### Digital health service hosting at different levels

##### National Health Data Centers (NHDC)

The national-level digital health services described in the Cloud-native OpenHIE-compatible National Digital Health Services section will be hosted in four prefabricated data centers [26] located in the capital Lilongwe (in the center), Blantyre (in the southwest), Zomba (in the southeast) and Mzuzu (in the north). Each data center will implement a stack of three cloud layers:

1. *Infrastructure-as-a-service (IaaS) layer:* OpenStack [27] will run on physical servers to instantiate virtual machines, manage computing/networking resources and offer site-to-site replication.
2. *Platform-as-a-service (PaaS) layer:* Ubuntu Linux Server [28] will be used as the operating system; Kubernetes [29] will be used as the containerized software manager, while a trusted Edge Computing platform will be used to establish T-REEs for providing secure communication and information isolation among the container pods.
3. *Software-as-a-service (SaaS) layer:* National digital health services will run as containerized software applications orchestrated by Kubernetes and protected by T-REEs.

High availability and automatic scaling of the national digital health services will be guaranteed by the horizontal pod autoscaling (HPA) process provided by Kubernetes and the replication process supported by OpenStack.

##### MaHIS at district and regional levels

The MaHIS (Malawi Health Information System section) will run on computing servers installed in district and regional hospitals. Kubernetes will be used to orchestrate the digital health applications running in container pods while Zero-Trust Secure Communication will be used to provide secure communication among the computing servers.

##### Health information points of service at community level

Currently, the iCHIS is implemented using the DHIS2 Android Capture App (Integrated Community Health Information System section). Individual patient’s and case-based data are captured in ADX data format [30] using Tracker [31]. When the T-NDHII is deployed, iCHIS will be hosted in a rugged portable computer running tMIG and FHIR/DICOMweb servers and connected with various digital healthcare devices necessary for running a community clinic (Principle 1: use of digital healthcare devices section).

#### Cloud-native Management of Trusted Medical Information Guards and Containerized Applications

The containerized software running in the OpenHIE-based NDHIS can be managed using the open-source tools available on the Cloud Native Computing Foundation (CNCF) Landscape [32]. Following is a list of tools to be installed in the National Health Data Centers (NHDC) for managing the tMIGs, the Edge/Cloud Computing servers and the containerized software applications running on them.

##### tMIG infrastructure management

In T-NDHII, OpenHIM will be deployed as a distributed interoperability layer with its core modules installed in every tMIG. Together, the OpenHIM cores will form a peer-to-peer overlay network that connects every tMIG in a community-level facility to a primary tMIG in its upstream district/regional hospital as well as one or more secondary tMIG(s) in the other hospitals as backups. Similarly, the tMIG in every district/regional hospital will connect to the tMIG in one of the four data centers as its primary upstream peer and the tMIG of the other data centers as its secondary peers. These primary and secondary peer connections will ensure the high availability of OpenHIM routes and the survivability of the entire OpenHIE structure.

##### Software container management

The health information services provided at the district/regional/national level as well as the tMIG and OpenHIM components are all containerized software modules. They can be deployed and replicated using the Kubernetes container orchestrator [29]. The standard K8s implementation will be deployed in the data centers while the lightweight K3s implementation will be used in the community/district/regional-level facilities.

A private container registry will also be hosted among the data centers. This CNCF compliant registry will maintain all the containerized software running in the NDHIS.

##### Cloud-native system management

The following three management and monitoring services will be used to run the CNCF compliant T-NDHII infrastructure:

***Certificate authority:*** All tMIGs and Edge/Cloud servers shall have public key-based credentials. The equipment supplied by Taiwan vendors will all have credentials issued by the official Taiwan Certificate Authority (TWCA). All T-REEs instantiated in the computing equipment shall have public key-based credentials issued by a CNCF graduated certificate manager such as the Vault [33]. The Malawi MoH shall choose an internationally accredited certificate authority as the root CA.

***System state monitor:*** Status and performance of Kubernetes Pods and the software containers running in them will be monitored continuously using CNCF graduated monitors such as Prometheus [34] and Grafana [35].

***System event auditor:*** System-level events on both physical equipment and virtualized platforms will be logged using CNCF graduated tools such as Grafana Loki [36]. Real-time event alerts can be issued through email and social media according to user-defined alert policies.

## RESULT

### OpenHIE-based NDHIS

**Figure 5:**
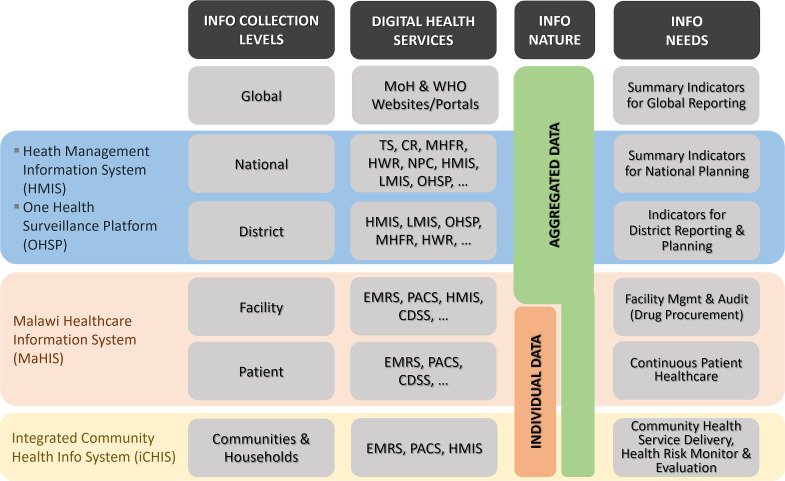
Organizational structure of Malawi NDHIS

Malawi NDHIS is the digital health information system deployed onto T-NDHII to implement the country’s digital health services and policies [4]. As shown in Fig. 5, it consists of three levels: community, patient/facility and district/national levels.

At the community level, the iCHIS is used to capture individual-level health data for use in the upper NDHIS levels (Integrated Community Health Information System section).

At the patient/facility level, Malawi Healthcare Information System (MaHIS) is a comprehensive and integrated hospital-wide information system to provide services such as the National Electronic Medical Records System (NEMRS) to all health service delivery departments, Picture Archiving and Communication System (PACS) for medical/clinical radiology applications, the Health Management Information System (HMIS) for facility management and use the DHIS/ADX data format for upward reporting.

At the district/national level, all national digital health services, including the registry services and the business domain services mentioned in the Cloud-native OpenHIE-compatible National Digital Health Services section, will be hosted in the NHDC data centers.

In NDHIS, individual’s health data are captured at the community and facility levels to support continuous care for that individual. These data will be aggregated at district, regional, national and even global levels for management, planning, resource allocation and health risk surveillance.

### Integrated Community Health Information System (iCHIS)

The first-generation iCHIS POS is built upon a DHIS2 Tracker App [37] and runs on Android tablets. The current iCHIS has twelve modules performing community-level register, surveillance, curation health services and HMIS functions. The system will cover all community healthcare services including curative, preventive, rehabilitative and general health management.

**Figure 6:**
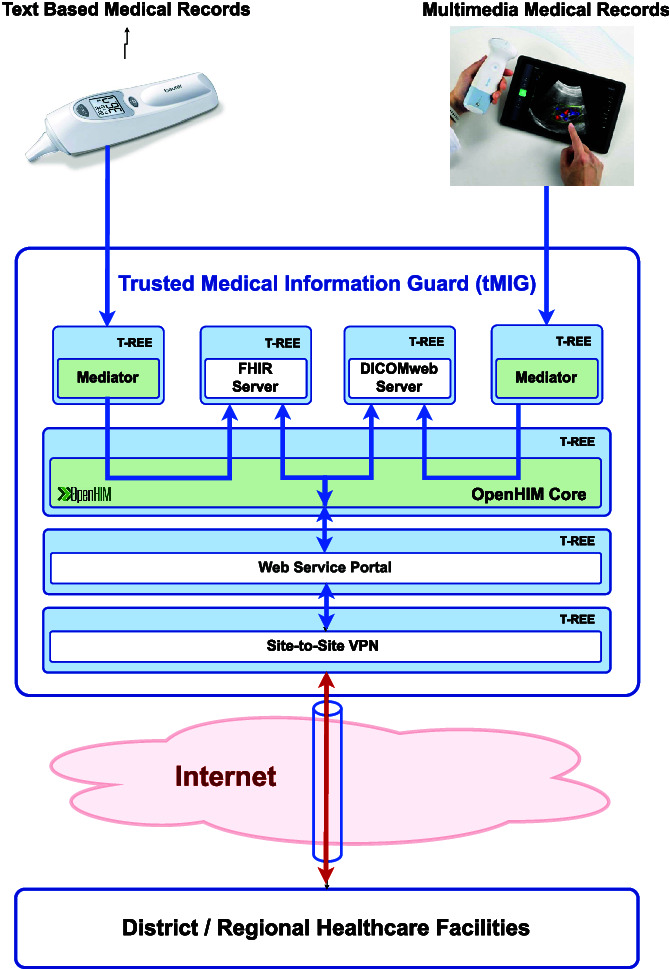
A second-generation iCHIS POS protected by a mini-tMIG as its gateway

The second-generation iCHIS POS will be equipped with a mini-tMIG (Fig. 6). Beside of running DHIS2 Tracker, it will be connected with handheld digital healthcare devices to capture patient’s medical data (Principle 1: use of digital healthcare devices section), store those data in a HAPI FHIR server and a DICOMweb server built into the tMIG and upload them to a facility-level MaHIS when a stable mobile communication link can be established. The new iCHIS POS with portable power sources can be installed in the rural clinics or carried in cars across the countryside.

### Malawi Health Information System (MaHIS)

**Figure 7:**
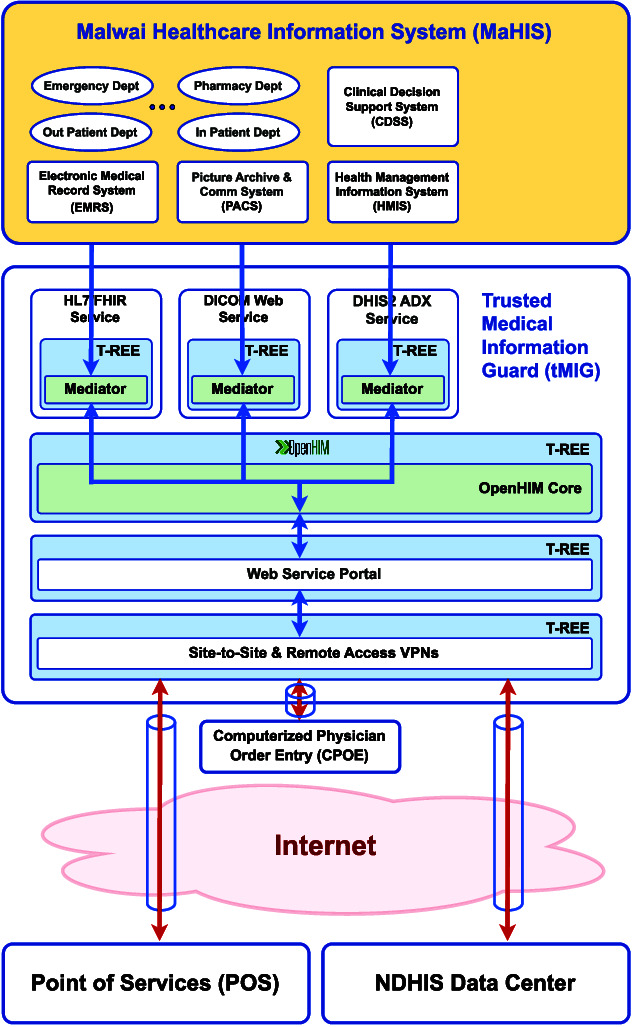
A MaHIS deployment protected by a tMIG as its frontend

MaHIS (Fig. 7) is a hospital-wide digital health information system that enables healthcare professionals in different departments to access patients’ electronic medical records, enter diagnostic decisions and order test or treatment procedures on their Computerized Provider Order Entry (CPOE) consoles. MaHIS is an integrated system as it maintains a single electronic medical record for each patient. It is also a unified system as it uses three standard protocols to manage digital health data for every patient: (i) HL7/FHIR for electronic medical records; (ii) DICOMweb for medical images; (iii) DHIS2 with ADX format for health management information.

In T-NDHII, the community-level iCHIS will be connected to the facility-level MaHIS, which will in turn be connected to the National Health Data Centers (NHDC), all through a peer-to-peer network of tMIGs equipped with OpenHIM Cores (Trusted Medical Information Guards section). This distributed interoperability layer will enable individual and aggregated health data to be shared across all three NDHIS levels.

### Cloud-native OpenHIE-compatible National Digital Health Services

The MoH DHD has been developing the following national-level digital health services as OpenHIE service components. They are currently deployed in the server rooms on the government premises and will later be hosted in the National Health Data Centers (NHDC).

#### Registry services

1. ***Terminology service (TS):*** Currently, Malawi uses the Open Concept Lab (OCL) terminology management system [38]. DHD is also working with OCL and Columbia International eHealth Laboratory (CIEL) to bridge the local terms with the international standards.
2. ***Client registry (CR):*** Malawi uses the CDC/PEPFAR Demographic Data Exchange (DDE) service to issue unique health identification numbers (UHIDs) to its patients. The UHIDs are now printed as QR code stickers. MoH will work with the National Registration Bureau to integrate the civil registration and vital statistic system (CRVS) with the UHID system.
3. ***Facility registry (FR):*** The Zipatala master health facility registry has been fully developed and is now available for public use [39].
4. ***Health worker registry (HWR):*** The integrated human resource information system (iHRIS) developed by MoH will become the single source of all health worker data. MoH will issue Healthcare Worker identities (HWIDs) with the National Registration Bureau for enforcing access control to the NDHIS.
5. ***Product catalogue:*** The MoH Health Technical Support Services (HTSS) department is driving the standardization of medical commodity and supply codes based on GS1-GTIN [40].

#### Business domain services

1. ***Logistics management information systems (LMIS):*** Malawi built its LMIS upon OpenLMIS. This system will be linked to the point-of-service system as a part of the NDHIS development.
2. ***Health management information system (HMIS):*** Malawi developed a mature HMIS based on DHIS2. This system can now gather health statistical data from all departments and programs.
3. ***One health surveillance platform (OHSP):*** Developed by DHD for timely outbreak surveillance and emergency response, OHSP is a component of a nation-wide electronic integrated disease surveillance and response (eIDSR) system. DHD is also developing an event-based surveillance (EBS) function to capture potential public health issues.

## USE CASES

T-NDHII promises to support secure health information interchanges among national data centers, district hospitals and community clinics as well as enabling privacy-preserving health information sharing with international collaborators for clinical research and AI model training. The following two use cases demonstrate the potential use for these purposes.

### Guarded internet access of hospital information system from authorized medical clinics

The provision of reliable, secure and privacy-preserving health information access through a tMIG was first demonstrated in 2023 at Taiwan Pingtung Christian Hospital (PTCH), a medical outpost of Norwegian Mission Alliance [41].

This pilot project aims at allowing authorized off-site medical clinics to refer their patients to PTCH and track their treatments online. A CPOE console installed in an affiliated medical clinic can authenticate itself with a USB dongle. An authorized user can then log onto the CPOE to establish a secure internet connection with the PTCH tMIG. Depending on the user’s roles, he/she can access certain patient records maintained in the PTCH hospital information system.

**Figure 8:**
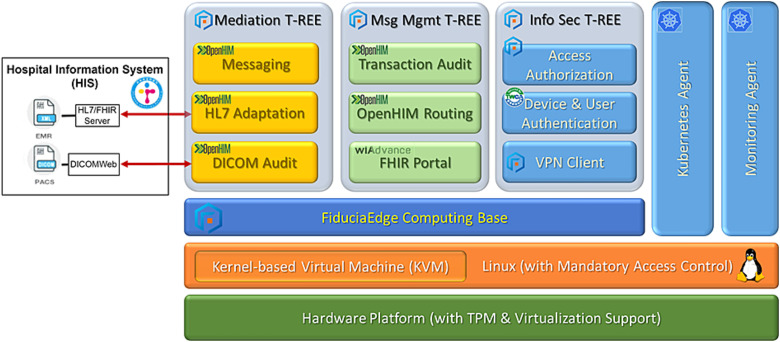
Functional composition of tMIG used in the PTCH pilot project to permit external access of PTCH HIS by authorized community medical clinics

Figure 8 shows the security, routing and mediation services deployed in the tMIG. Each service is implemented as a micro-service encapsulated in an OCI-compatible software container, certified by the Taiwan official certificate authority (TWCA) [42], and runs in a T-REE. These micro-services can be classified in the following three categories:

1. *Information Security Services,* which include virtual private networking (VPN) through the public internet, CPOE system authentication based on the USB security dongle, user authentication and intranet access control.
2. *Message Management Services,* which include message routing and transaction auditing services offered by the OpenHIM Core. In addition, a web portal server developed by WiAdvance [43] was installed to allow users to interact with the tMIG using a web browser.
3. *Message Mediation Services,* which include OpenHIM mediators used to monitor, convert and control message exchanges between the CPOE console and the PTCH HIS. A mapping mediator [44] and an RFC3881 to DICOM audit message mediator [45] are used in this pilot project.

### Adaptive illness severity prediction based on privacy-preserving machine learning

Current strategies for treating childhood pneumonia in Sub-Saharan Africa follow the standard procedures of illness identification and risk score-based severity assessment by primary care providers before the severe cases are referred onto hospitals. The traditional approach to establish the risk scores is based on research studies conducted among certain populations in secondary or tertiary healthcare facilities and may take years to complete. This approach greatly limits its applicability to different target populations and its adaptability to the changing circumstances [46]. In the BIOmarkers TO Predict pneumonia (BIOTOPE) (Phase I) project [47], a research team from University Colleges of Galway, Cork and Dublin in Ireland developed the machine learning (ML) techniques that can update the risk scores of childhood pneumonia continuously based on the routine healthcare data collected by the primary care providers. These ML-adapted risk scores have been demonstrated to be more accurate in predicting the severity of patients’ illness [48].

In BIOTOPE (Phase II), the research team is working with Malawi MoH to deploy their ML algorithms in NDHIS. These algorithms will be integrated with iCHIS to help community health workers to identify children at risk of severe illness. At the same time, the ML algorithms are retraining the risk scores to achieve higher accuracy and become more adaptive to emerging pathogens.

**Figure 9:**
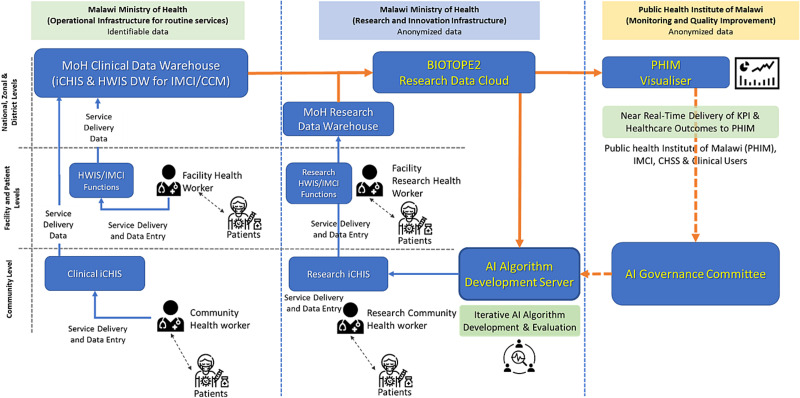
Process topology and data flows in the prize phase of BIOTOPE-II

As shown in Fig. 9, BIOTOPE-II will gather routine healthcare data from both Clinical and Research iCHIS. Through mutually-authenticated and secured internet communications, the iCHIS will transfer their data to the corresponding data warehouses. Through a tMIG, the private BIOTOPE Research Data Cloud can access relevant data in the data warehouses after obtaining proper authorization and then process those data in a T-REE instantiated in the Research Data Cloud. The data reside in the warehouses will be managed by Malawi MoH. Data confidentiality, integrity and sovereignty will be fully protected by the tMIG and the T-REE. Finally, the ML program codes must be certified by a trusted third party before they can be run in the BIOTOPE Research Data Cloud.

BIOTOPE-II will also employ a two-pronged approach to protect the ML models from potential contamination by inaccurate data.

1. Each data element is specified to fall within a set range. If a data element sent to the ML training algorithm is out of range, it will not be used, and its occurrence will be reported. This report will trigger a follow-up procedure that will check the accuracy of the measuring device.
2. When the ML algorithm makes a new recommendation that falls outside a fixed tolerance, the AI governance committee will review the case. The quality of the data used by the ML algorithm will be reported to the AI governance committee to assist them with their decision making.

The BIOTPE-II strategy of adjusting illness severity classification criteria based on the healthcare data collected continuously from the patient population promises a novel approach to clinical risk prediction. It also demonstrates the necessity of data privacy and sovereignty protection in AI model training and decision making.

## DISCUSSION

### T-NDHII rollout

**Figure 10:**
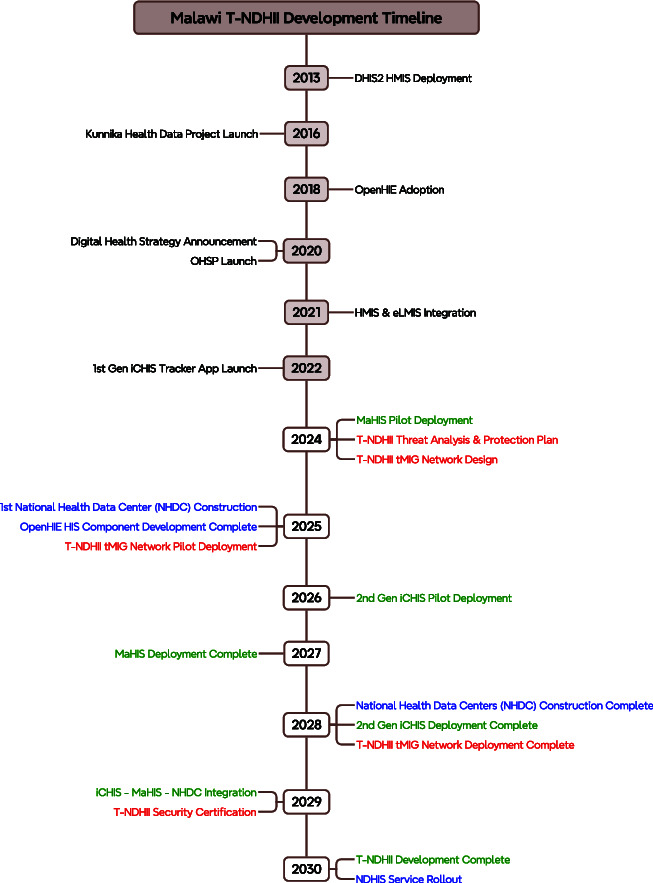
Malawi T-NDHII development timeline

Figure 10 shows the T-NDHII development timeline. Activities occurred before 2024 were mentioned in History of digital healthcare in Malawi Section . The events that will occur during 2024–2030 are color-coded to show the plan to roll out the security infrastructure (in red), the Edge equipment and services (in green) and the Cloud data centers and services (in blue). The ultimate goal is to have an operating infrastructure by 2030.

#### Security infrastructure

The development of T-NDHII security infrastructure begins with a thorough threat analysis and protection strategy planning. The preliminary findings and proposed strategy were reported in this paper. This study will continue with the detailed design of the tMIG network for connecting the iCHIS with the MaHIS and ultimately with the NHDC. This network will become the backbone of T-NDHII. A pilot deployment of the tMIG network between district-level MaHIS and the NHDC will start in early 2025. Once this pilot network becomes operational, it will be expanded to connect the second-generation iCHIS with lower-level MaHIS. By the end of 2028, the tMIG Network will connect communities and facilities covering at least 70% of the country’s population.

In early 2030, the IT equipment and operation in the regional and national facilities shall pass ISO27001 so that T-NDHII can go online by the end of 2030.

#### Edge systems and services

A pilot deployment of MaHIS has begun in selected district hospitals in early 2024. Once they are tested, MaHIS will be deployed in all public healthcare facilities in the country. All district and regional hospitals should have it installed by the end of 2027.

The first-generation iCHIS, which was built upon a DHIS2 Tracker app, was launched in December 2022 in three districts, Balaka, Machinga and Salima, to service approx. 644 000 people [49]. The Tracker app will be connected to the MaHIS pilot by the end of 2024. The second-generation iCHIS equipped with digital healthcare devices will start its deployment in 2026 and be connected directly to MaHIS through tMIGs. By the end of 2028, iCHIS and MaHIS combined shall serve more than 70% of the population. Such development plan echoes the proposed solution from WHOAFRO [50].

#### Cloud systems and services

At the national level, MoH plans to complete the development of the OpenHIE-compatible National Digital Health Services mentioned in the Cloud-Native OpenHIE-Compatible National Digital Health Services section by the end of 2025.

MoH will also start the construction of the first National Health Data Center (NHDC) in 2025 with the support from the World Bank [26]. Four data centers will be built by the end of 2028. This will ensure the necessary computing power will become available in time.

### Regulation compliance

Malawi Communications Regulatory Authority (MACRA) is the regulatory body that oversees the protection of personal digital data. The responsibility of monitoring NDHIS compliance to health data privacy protection regulations is assigned to the Compliance Office under the MoH DHD. Their compliance officers will report any regulation breach by in-house and externally-developed systems to the DHD Compliant Office and, when necessary, to MACDA. In addition, MACRA, together with MoH, will appoint an independent agency to review the security practice of NDHIS and provide recommendations for improvement.

All the containerized software running on a tMIG including OpenVPN, OpenHIM, its mediators and the web service portal will be verified by a certified testing company and then digitally signed by an internationally accredited certificate authority to certify that these software modules are free of any critical security vulnerability above CVE Level-7.

### UHC fulfilment

Digital health interventions, such as those employed in Nigeria, demonstrate the potential in using decentralized patient record systems to improve health data quality and support UHC [51]. Similarly, Malawi T-NDHII will facilitate the integration of individual and aggregated health data across different health domains while enhancing data accuracy, security and availability using the state-of-art technology. Besides developing T-NDHII, DHD is putting together an organization development plan aligned with the findings from these relevant studies [40, 52].

### Management and governance

Several challenges including inadequate healthcare worker training, poor internet connectivity in rural areas and fragmentation of digital health services were mentioned in Challenges in existing system and practice section. These issues cannot be resolved through technology. MoH is addressing them through sustainable infrastructure investment, worker training and robust governance mechanism development. Furthermore, DHD has established the Digital Health Technical Working Group to receive user feedback and stakeholder suggestions in order to improve the NDHIS architecture and its operation. All suggestions will be reviewed by the Senior Digital Health Technical Advisors and devise proper responses.

## CONCLUSION

This paper introduced the design of a scalable and trustworthy digital health information infrastructure that is based on the OpenHIE architecture and conforms to the Kubernetes-based Cloud Native Computing framework and the Trusted Computing practice. The scalability and trustworthiness of this infrastructure hinge on the use of a single security element, *the trusted Medical Information Guard (tMIG)*, which protects information transport and transformation through the Open Health Information Mediators with Multiple Independent Levels of Security (MILS) and Zero-Trust Network Security Architecture (ZTA). This tMIG security element can be implemented using containerized software running in the Trusted Rich Execution Environments (T-REEs). A peer-to-peer network of tMIGs orchestrated using Kubernetes can offer high-assurance protection of data availability, security and privacy to the computing and communication equipment attached to this network.

By adopting the proposed T-NDHII, Malawi strives to improve the accuracy, accessibility and security of its health data as stated in DHS Objectives 4–7. The success of this endeavor will depend on collaborative partnership, effective governance and sustained investment in capacity building and infrastructure development. This holistic approach will empower Malawi to achieve a sustainable, reliable and secure national digital health system that will enable the country to achieve the UN SDGs in good health and well-being.

Continuous study should be conducted to evaluate the long-term impact of these digital health interventions and incorporate additional strategies to ensure their cost effectiveness and ease in use.

## Data Availability

The data underlying this article will be shared on reasonable requests to the corresponding author.

## Footnotes

1 A computing system is *trustworthy* if it has the inherent property of information reliability, security, privacy and integrity.

2 The DHS objectives and empowering strategies were abridged for the sake of brevity.
